# Going to College

**Published:** 1873-10

**Authors:** 


### GOING TO COLLEGE.

THE cost of a College education is an item of considerable interest to many families. The necessary expenses a year at Oxford, England, are $250.

Williams' College, Mass., founded 1793, $200.

University of Virginia, founded by Jefferson, in 1816, at Charlottesville, Virginia, $300.

Cornell University, at Utica, N. Y., $350.

At Harvard, near Boston, founded in 1638, the average expenses are $400.

Yale, chartered 1701, $500,

In these items are not included clothing, travelling and personal indulgences.

The healthiest method of getting an education is to work out of doors a part of every day and study another part. Several attempts have been made in this direction, but all have failed for want of proper method in the arrangement of the hours of study and labor. If these were judiciously regulated there can be no reasonable doubt that the student would graduate quite as soon, and quite as creditably, with much more constitutional stamina, and that much more he would be a vigorous thinker and an efficient worker.

The student should be required to rise an hour before sunrise, so as to be able to dress and take breakfast by the time it is light enough to read without artificial light and without straining the eyes; a breakfast of cold bread and butter, a cup of hot drink and a piece of meat, nothing else whatever; or he might take boiled or baked potatoes, or buckwheat cakes instead of the meat, occasionally, for a change. Of a plain breakfast of this kind there would be no temptation to take too much. Then walk half an hour in the open air, and give five hours for study; take a hearty dinner, and work until dark. After dark make the recitations, and be in bed at nine o'clock in summer, and not later than ten in winter. This will prevent injuring the eyes by too great use of them by artificial light. The supper should be made at twilight, so as to lose none of daylight for working purposes.

Some of the young gentlemen at Cornell University are able to pay a considerable portion of their expenses by labor of various kinds, and for which they deserve credit. They are thus laying that foundation of personal independence, and self-reliance, and self-denial which will be worth more to them in the battle of life than any amount bestowed in charity or derived from inheritance.

The philosophy of this programme for study, and labor, and recitation will bear investigation, and will be suggestive to thoughtful men.

